# Increased Mortality in Mice following Immunoprophylaxis Therapy with High Dosage of Nicotinamide in *Burkholderia* Persistent Infections

**DOI:** 10.1128/IAI.00592-18

**Published:** 2018-12-19

**Authors:** Sofiya N. Micheva-Viteva, Brittany N. Ross, Jun Gao, Samantha Adikari, Pengfei Zhang, Judith R. Mourant, Terry H. Wu, James H. Werner, Alfredo G. Torres, Elizabeth Hong-Geller

**Affiliations:** a Bioscience Division, Los Alamos National Laboratory, Los Alamos, New Mexico, USA; b Department of Microbiology and Immunology, University of Texas Medical Branch, Galveston, Texas, USA; c Center for Integrated Nanotechnologies, Los Alamos National Laboratory, Los Alamos, New Mexico, USA; d University of New Mexico Health Sciences Center, Department of Internal Medicine, Albuquerque, New Mexico, USA; University of California San Diego School of Medicine

**Keywords:** *Burkholderia*, antimicrobial resistance, bacterial persistence, microfluidics, nicotinamide, *relA*

## Abstract

Bacterial persistence, known as noninherited antibacterial resistance, is a factor contributing to the establishment of long-lasting chronic bacterial infections. In this study, we examined the ability of nicotinamide (NA) to potentiate the activity of different classes of antibiotics against Burkholderia thailandensis persister cells. Here we demonstrate that addition of NA in *in vitro* models of B. thailandensis infection resulted in a significant depletion of the persister population in response to various classes of antibiotics. We applied microfluidic bioreactors with a continuous medium flow to study the effect of supplementation with an NA gradient on the recovery of B. thailandensis persister populations. A coculture of human neutrophils preactivated with 50 µM NA and B. thailandensis resulted in the most efficient reduction in the persister population. Applying single-cell RNA fluorescence *in situ* hybridization analysis and quantitative PCR, we found that NA inhibited gene expression of the stringent response regulator *relA*, implicated in the regulation of the persister metabolic state. We also demonstrate that a therapeutic dose of NA (250 mg/kg of body weight), previously applied as immunoprophylaxis against antibiotic-resistant bacterial species, produced adverse effects in an *in vivo* murine model of infection with the highly pathogenic bacterium Burkholderia pseudomallei, indicating that therapeutic dose and metabolite effects have to be carefully evaluated and tailored for every case of potential clinical application.

## Abstract (precis)

Bacterial persistence, known as noninherited antibacterial resistance, is a factor contributing to the establishment of long-lasting chronic bacterial infections. In this study, we examined the ability of nicotinamide (NA) to potentiate the activity of different classes of antibiotics against Burkholderia thailandensis persister cells.

## INTRODUCTION

There is an urgent need to develop novel antimicrobial therapies to combat the rapid spread of antibiotic-resistant bacteria. In addition to the well-studied evolution of genetic resistance to antibiotics, bacteria can enter a dormant metabolic state, termed bacterial persistence or noninherited antibacterial resistance, in which cells undergo phenotypic changes that render them refractory to virtually all known classes of antibiotics, thus leading to a recurrence of chronic infections and biofilm formation (1–3). Bacterial persistence has been described more recently as an evolutionarily conserved mechanism that serves as community life insurance against a hostile environment (4). Multiple redundant pathways are thought to contribute to the establishment of the persister metabolic state, including accumulation of the stringent response signaling nucleotide (p)ppGpp, the activity of the Lon protease, and toxin/antitoxin modules (5, 6). In addition, mRNA endonucleases, including RelE, MazF, and YafQ, and phosphorylation of the glutamyl-tRNA synthetase GltX have been attributed to the establishment of persistence (7, 8). Given the multiple mechanisms that regulate persistence, therapeutic development to eradicate persister cells remains particularly challenging.

Several promising strategies that target bacterial persistence have been reported. Since persisters are thought to have reduced metabolic activity, stimulation of metabolic pathways may reactivate persister growth, thus making them more susceptible to antibiotic treatment. In support of this hypothesis, addition of metabolites that fuel glycolysis, such as glucose, mannitol, and fructose, has led to an increase in aminoglycoside antibiotic uptake through stimulation of the proton motive force and subsequent enhanced killing of persisters in Escherichia coli (9). Persisters have also been depleted by supplementation of conventional antibiotics with novel antibacterial classes, such as acyldepsipeptides (ADEPs) (10). ADEPs deregulate ClpP protease activity, leading to uncontrolled protease degradation, inhibition of cell division, and, eventually, cell death (11, 12). However, ADEPs have very limited activity against Gram-negative bacteria. *clpP* mutants resistant to ADEP arise at a high frequency because ClpP is not essential for the bacterial life cycle (11).

To further investigate metabolite-mediated strategies to target bacterial persistence, we examined the ability of nicotinamide (NA) to potentiate the activities of different classes of antibiotics against persisters of Burkholderia thailandensis CDC2721122, a clinical isolate from a pleural wound. NA, also known as vitamin B_3_, is a precursor of nicotinamide adenosine dinucleotide (NAD), a key cofactor for enzymes that play a role in oxidative phosphorylation. We hypothesized that NA could potentially kick-start energy metabolism to a level sufficient for bacteria to exit the persistence state and, thus, recover sensitivity to antibiotics. In addition, NA has been shown to stimulate the killing of Staphylococcus aureus and Citrobacter rodentium in murine models (13, 14). We demonstrated a significant depletion of the persister population in response to various classes of antibiotics when we applied NA in *in vitro* models of B. thailandensis infection. However, the use of NA in an *in vivo* murine model of infection with Burkholderia pseudomallei, a highly pathogenic select agent that is closely related to B. thailandensis and causes the infectious disease melioidosis, produced unexpected adverse effects, indicating that NA will likely not be therapeutically effective when supplied at a high dose. Here, we raise awareness that therapeutic strategies previously shown to be successful in *in vivo* systems of opportunistic bacterial infections may be detrimental for the treatment of infections caused by pathogens that target innate immune responses.

## RESULTS

### NA potentiated the bactericidal effect of various antibiotics against B. thailandensis persisters.

**FIG 1:**
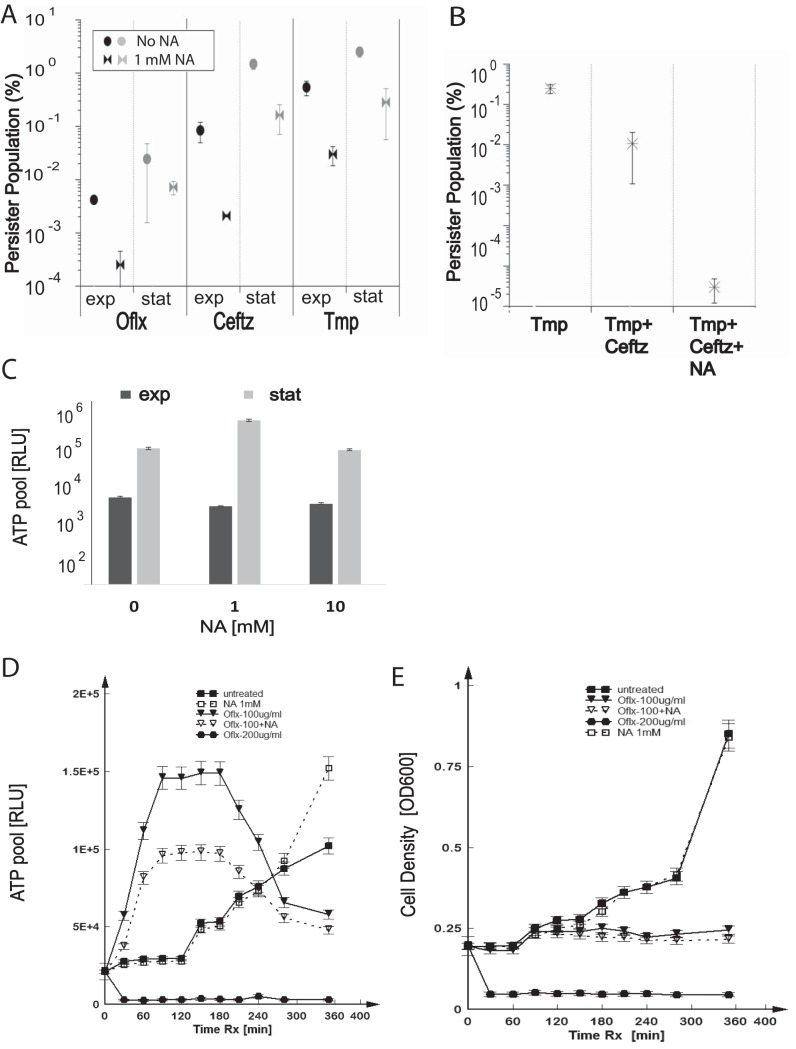
NA enhances B. thailandensis sensitivity to antibiotics and stimulates persister metabolism *in vitro*. (A) B. thailandensis CDC2721122 cultures at exponential (exp) or stationary (stat) phase were treated with bactericidal concentrations of Oflx (100 µg/ml), Ceftz (150 µg/ml), and Tmp (250 µg/ml) in the presence or absence of 1 mM NA for 24 h. The size of the persister populations was determined as the percentage of the number of CFU per milliliter surviving the antibiotic treatment relative to the bacterial count prior to antibiotic exposure. Averages ± standard deviations were calculated from four independent experiments. (B) B. thailandensis cultures at stationary phase were treated with Tmp (250 µg/ml), Tmp (100 µg/ml)-Ceftz (50 µg/ml), and Tmp (100 µg/ml)-Ceftz (50 µg/ml)-NA (1 mM) for 24 h. Persister populations were calculated as described in the legend to panel A, and statistical analysis was performed on data obtained from three independent experiments. (C) Bacterial cultures were grown in LB medium supplemented with 1 mM NA, 10 mM NA, or no NA. The total ATP pool for B. thailandensis in the exponential and stationary phases was measured as the number of relative light units (RLU). A representative result of five independent experiments is shown, and statistics were determined from three replicates from the same experiment. (D, E) B. thailandensis cells were treated with 100 µg/ml Oflx, 200 µg/ml Oflx, and/or 1 mM NA, as indicated, at exponential phase. Samples were analyzed at 30-min intervals for ATP content (D) and cell density (E) by measuring the luminescence and OD_600_, respectively. A representative result of five independent experiments is shown. Averages ± standard deviations were calculated from three replicates per experimental condition. Rx, treatment.

We observed that the size of the persister population within planktonic B. thailandensis CDC2721122 cultures varied depending on the class of antibiotic used. Ofloxacin (Oflx), a fluoroquinolone that targets DNA replication, was 50-fold and 100-fold more effective in killing persisters than ceftazidime (Ceftz) and trimethoprim (Tmp), respectively (Fig. 1A). We also noted that *Burkholderia* cells entered persistence at a higher rate than previously studied organisms, up to 1% in stationary phase compared to 0.1% in E. coli (Fig. 1A and B) (6, 15, 16). Consistent with the bacterial stringent response to nutrient limitation, the size of the B. thailandensis persister populations was ∼10-fold higher in stationary phase than when cells were exponentially dividing (Fig. 1A and B). Addition of 1 mM NA prior to antibiotic treatment decreased persisters by >10-fold in both exponential- and stationary-phase B. thailandensis cultures treated with each of the three antibiotics, indicating that NA can potentiate the antibiotic-mediated killing of persisters (Fig. 1A).

Ceftz, a β-lactam antibiotic, has been an effective therapy for melioidosis, the infectious disease associated with B. pseudomallei infection. Two regimens are commonly prescribed for initial treatment of severe melioidosis: (i) Ceftz monotherapy and (ii) Ceftz in combination with trimethoprim-sulfamethoxazole (Tmp-SMX) (17). While the combination therapy has not been reported to have an advantage over the monotherapy in cases of acute melioidosis, it is possible that the Tmp-SMX component could potentiate the Ceftz treatment by reducing *Burkholderia* persisters. To test this *in vitro*, we treated B. thailandensis with Ceftz or a combination of Ceftz and Tmp. During Ceftz and Tmp monotherapy, persisters were present at 0.1% and 0.3%, respectively, at exponential phase and 1% and 2%, respectively, at stationary phase (Fig. 1A). Upon combination treatment with Ceftz and Tmp, we registered a >10-fold reduction in the percentage of persister survival compared to that during drug monotherapy at the stationary phase (Fig. 1B). Addition of 1 mM NA prior to the combination antibiotic therapy resulted in a >10,000-fold depletion of persisters compared to the number during monotherapy with Tmp (Fig. 1B).

Given the role of NA as a precursor of the key metabolite NAD in the oxidative phosphorylation pathway, we measured the total ATP pool in B. thailandensis bacteria treated with various concentrations of NA. We detected a 2-fold increase in the ATP pool in stationary-phase cells treated with 1 mM NA compared to that in cultures grown in the absence of NA. We did not see a similar increase upon addition of 10 mM NA. NA did not boost ATP generation in exponentially dividing cells (Fig. 1C).

To further examine the effect of NA on the antibiotic treatment of B. thailandensis persisters, we simultaneously measured the ATP pool and cell density in B. thailandensis bacteria treated with a 1× minimal bactericidal concentration (1× MBC) of Oflx (100 μg/ml). In general, the ATP content is expected to linearly correlate with the cell density in exponentially dividing cells. Surprisingly, we consistently registered a burst in ATP production in the first 3 h of B. thailandensis treatment with 1× MBC of Oflx, while the cell density was observed to stay relatively flat (Fig. 1D and E, Oflx at 100 μg/ml). This effect was not observed when cells were treated with 2× MBC of Oflx (Fig. 1D and E, Oflx at 200 μg/ml), which is consistent with cell death at this bactericidal concentration and which was concurrent with a decrease in cell density. Addition of 1 mM NA to bacterial cultures treated with 1× MBC Oflx resulted in a 2-fold reduction of ATP levels compared to those after treatment with 1× MBC alone, although the ATP pool remained 2.5-fold higher than that in the untreated, exponentially dividing cells (Fig. 1D, Oflx at 100 μg/ml plus NA). The observed ATP burst and decrease with NA supplementation was also observed in cells treated with 1× MBC of meropenem (Mpm), indicating that the observed effect is independent of the antibiotic mechanism of action (see Fig. S1 in the supplemental material). These results suggest that suboptimal bactericidal antibiotic concentrations can stimulate the ATP production that may be needed to fuel metabolic pathways that can induce the onset of persistence. Addition of NA resulted in the partial rescue of the enhanced ATP production, which may have contributed to the reduction in persistence. Thus far, the data indicate that use of a combination of NA with different classes of antibiotics may be a viable approach for more effective treatment of pathogen infection.

### NA can increase the susceptibility of B. thailandensis persister cells to antibiotics.

**FIG 2:**
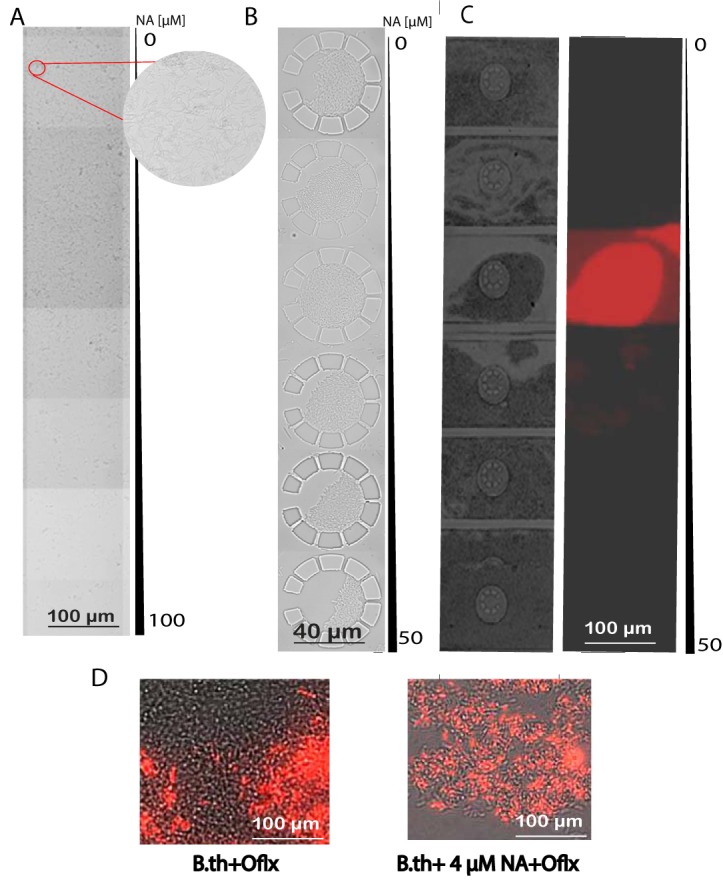
The antibiotic bactericidal effect against B. thailandensis persisters is dependent on the NA concentration. (A) The microfluidic device was infused with 100 µl B. thailandensis (B.th) culture (10^7^ cells/ml) in LB broth for 4 h without flow to achieve a universal distribution within the growth chamber. Syringes containing LB broth supplemented with 0 and 100 µM NA were attached to the left and right inlets, respectively, and the flow speed was adjusted to 5 µl/h. The bacterial density in the growth chamber was observed at 24 h postinfusion of NA with a Zeiss Axio Observer Z1 microscope. Shown is a bright-field image of the bacterial density built from 6 image fields in the same row taken from left to right (low to high NA concentration) across the width of the cell growth chamber. (B) Bright-field image of bacterial growth in the trapping chambers of the microfluidic device built from 6 image fields in the same row taken from left to right across the NA gradient. Syringes containing LB medium (left inlet) and LB supplemented with 50 µM NA (right inlet) were supplied into the growth chambers at 10 μl/h. B. thailandensis cells (100 μl, 10^7^ cells/ml) were infused through the inoculation inlet and incubated for 24 h in the NA gradient prior to replacement with LB medium containing 50 µg/ml Mpm for 48 h, followed by continuous flow with LB medium. (C) B. thailandensis growth in trapping chambers after 4 days of growth in antibiotic-free medium. PI (1 µg/ml) was added to the LB medium, and cellular viability was evaluated at an excitation wavelength of 520 nm and an emission wavelength of 610 nm. Tile images covering the same row of cell trapping chambers shown in panel B were acquired. A representative result of four independent experiments showing bright-field images (left) and fluorescence images at excitation and emission wavelengths of 520 and 610 nm, respectively (right), is shown. (D) B. thailandensis cultures (100 μl, 10^7^ cells/ml) were inoculated in microfluidic bioreactors infused with LB medium or LB medium supplemented with 4 μM NA. Following 18 h of bacterial growth in the bioreactors, 50 µg/ml Oflx was added to both experimental conditions. After 6 days of antibiotic exposure, the medium was replaced with antibiotic-free LB at a flow speed of 5 μl/h, and the microfluidic bioreactors were microscopically monitored for the recovery of bacterial growth. PI (1 µg/ml) was added to the LB medium at day 3 of antibiotic withdrawal. Shown are microscopy overlay images of bright-field images and fluorescence images at excitation and emission wavelengths of 520 and 610 nm, respectively, at 5 days after antibiotic withdrawal. A representative result of four independent experiments is shown.

We utilized a microfluidics platform to simulate the microscale environment and fluid dynamics of the host circulatory system that would be observed during bacteremia to determine the NA concentration at which *Burkholderia* persister populations become most sensitive to the antibiotic bactericidal effect (Fig. S2A). We generated an NA concentration gradient ranging from 0 to 100 μM by applying a microfluidic picoliter bioreactor as previously described (18). We observed that NA had little effect on bacterial growth at low concentrations (Fig. 2A, top) but started to exhibit a bacteriostatic effect at higher NA concentrations (∼50 to 100 μM), in which bacterial growth was inhibited (Fig. 2A, bottom). Interestingly, this effect lasted for 24 h with the continuous flow of medium supplemented with NA, after which the bacterial density became more uniform throughout the microfluidic channels, suggesting that *Burkholderia* is capable of overcoming the NA-mediated bacteriostatic effect (data not shown).

We also applied microfluidic devices containing cell trapper chambers that can corral bacteria into a partially enclosed area for concentrated bacterial growth during fluid flow. We observed inhibition of bacterial growth in these trappers at an estimated NA concentration of ∼50 μM (Fig. 2B, bottom trappers). We further studied the dosage effect of NA on *Burkholderia* sensitivity to antibiotics. B. thailandensis was preincubated in an NA concentration gradient for 24 h, exposed to either Mpm (50 μg/ml) or Ceftz (50 μg/ml) for 48 h, and then allowed to recover in NA- and antibiotic-free LB for 4 days. We observed that cells receiving ∼4 μM NA prior to antibiotic treatment exhibited low viability, as observed by high levels of propidium iodide (PI) staining of dead cells in the growth chamber of the microfluidic bioreactor, suggesting that persisters were susceptible to killing under these conditions (Fig. 2C, third panel from top). Pretreatment of cells with higher concentrations of NA (>10 μM) did not show the same effect. Metabolite concentrations in the microfluidic bioreactor were estimated relative to the concentration gradient of a colorimetric dye (Fig. S2B). We also confirmed the effects of 4, 16, and 25 μM NA by applying a single concentration of the metabolite in the bioreactor (Fig. 2D and data not shown). Taken together, our results indicate that there is a sweet spot of an optimal NA concentration of ∼4 μM that may switch persisters out of the dormant state and render them responsive to killing by antibiotics. Importantly, the NA effective concentrations reflected the scale of the bacterial growth environment: millimolar concentrations for milliscale bacterial cultures (Fig. 1) and micromolar concentrations for bacteria grown in the microfluidic channels (Fig. 2).

### NA reduces B. thailandensis persisters by inhibition of the bacterial stringent response regulator RelA and stimulation of the bactericidal effect in host neutrophils.

**FIG 3:**
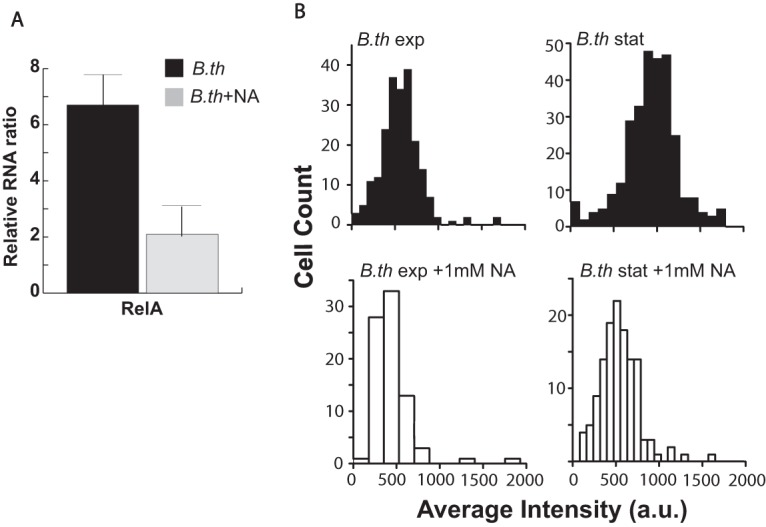
NA inhibits transcription of the stringent response regulator RelA in B. thailandensis. (A) Total RNA was isolated from exponential- and stationary-phase B. thailandensis grown in LB supplemented with 1 mM NA and subjected to RNA-to-Ct one-step RT-qPCR analysis. *relA* RNA levels relative to the gene expression levels in exponential-phase cells grown in LB with no NA supplementation were calculated. Shown are *relA* RNA ratios normalized to the level of the chaperone *dnaJ* as a sample loading control. Averages ± standard deviations were calculated from 3 independent experiments. (B) RNA-FISH histograms of *relA* fluorescent intensity distribution in single cells from exponential- and stationary-phase cultures of B. thailandensis in LB medium or LB medium supplemented with 1 mM NA. Cells were fixed and hybridized to 1 µM fluorescently labeled probes against *relA* transcripts. A representative result from four independent experiments is shown. a.u., absorbance units.

We investigated whether the NA-mediated decrease in *Burkholderia* persistence is linked to the activity of RelA, which regulates the concentration of the ppGpp alarmone in response to various environmental cues, including temperature change, nutrient limitations, and transition to stationary phase. Cellular accumulation of ppGpp initiates stress responses referred to as the “stringent response,” which contributes to the establishment of the persister metabolic state (19, 20). On average, the transcript levels of *relA* in stationary-phase B. thailandensis cells (≥1% persisters) were >6-fold higher than those in cells at the exponential phase of growth (≤0.1% persisters), as measured by quantitative PCR (qPCR). The *relA* transcript levels were reduced by ∼3-fold in stationary-phase cells upon addition of NA in the culture medium, indicating inhibition of the stringent response in B. thailandensis by NA (Fig. 3A).

We further investigated the effect of NA on *relA* gene expression at the single-cell level using RNA-based fluorescence *in situ* hybridization (RNA-FISH). Consistent with the qPCR results, *relA* gene expression was significantly higher in stationary-phase B. thailandensis populations than in exponentially dividing bacterial populations (Fig. 3B). Addition of NA to B. thailandensis cells at stationary phase inhibited *relA* expression compared to that in their untreated counterparts. Interestingly, RNA-FISH analysis revealed a very small population (≤0.01%) of B. thailandensis cells in the exponential growth phase with *relA* fluorescence intensities comparable to those in stationary phase, suggesting that these outliers may be stochastically overexpressing *relA* to cause a shift in these cells to persistence. Altogether, these results suggest that NA can inhibit the stringent response and may reduce the population of persisters that can survive antibiotic treatment.

To assess the therapeutic potential of NA in combination with antibiotic treatment, we employed the microfluidic bioreactors described earlier to grow B. thailandensis cultures over several days or weeks under continuous fluid flow and compared the time required for the repopulation of the growth chambers with bacteria surviving antibiotic treatment. Following 6 days of incubation with 50 μg/ml Mpm in the microfluidic device, B. thailandensis monocultures contained mostly dead cells, as determined by propidium iodine (PI) staining (data not shown). We next infused the microfluidics device with antibiotic-free medium to stimulate any persisters that remained recalcitrant to Mpm and observed the recovery of bacterial regrowth after 4 days of continuous culture (Table 1). A combination of 4 μM NA with Mpm treatment led to the recovery of the B. thailandensis persister population in 5 days, suggesting that NA reduced persister cell survival. We also observed bacterial recovery after 4 days following incubation with 400 μg/ml Tmp, after 5 days following incubation with 50 mg/ml Oflx, and after 10 days following incubation with 100 μg/ml Oflx. We consistently observed an increase in the number of days needed for B. thailandensis regrowth in the device when medium was supplemented with NA, regardless of the mechanism of antibiotic action (Table 1).

**TABLE 1:** Effect of antibiotic type, dose, and NA supplementation on the recovery time of *B. thailandensis* persister populations^*a*^

Experimental condition	Recovery time (days) in LB medium
Mpm (50 µg/ml)	4
Tmp (400 µg/ml)	4
Oflx (50 µg/ml)	5
Oflx (100 µg/ml)	10
Mpm (50 µg/ml) and 4 µM NA in medium	5
Mpm (50 µg/ml), neutrophils	7
Mpm (50 µg/ml), neutrophils, 4 µM NA in medium	7
Mpm (50 µg/ml), neutrophils, 25 µM NA in medium	12
Mpm (50 µg/ml), neutrophils pretreated with 50 µM NA, 4 µM NA in medium	15

a All experiments were independently repeated at least three times.

We further investigated B. thailandensis bacteria exposed to Mpm in the presence of human neutrophils, to add a physiologically relevant host immune function to the microfluidic bioreactor. Bacterial coculture with neutrophils at a 1:1 cell ratio resulted in a greater delay in bacterial regrowth following Mpm treatment, occurring at 7 days after antibiotic treatment. Addition of 25 μM NA to B. thailandensis-neutrophil cocultures treated with Mpm led to an even longer recovery time, occurring at 12 days after antibiotic treatment. Notably, a combination of Mpm with 4 μM NA and neutrophil coculture took 7 days for bacterial recovery, similar to the findings for coculture in the absence of NA. This observation suggests that higher NA doses may be required for stimulation of the neutrophil bactericidal function. In support of this hypothesis, recovery of bacterial growth was not observed until 15 days after antibiotic treatment when neutrophils were preincubated with 50 μM NA for 24 h prior to coculture with *Burkholderia* and subsequent addition of 4 μM NA in the growth medium (Table 1).

**FIG 4:**
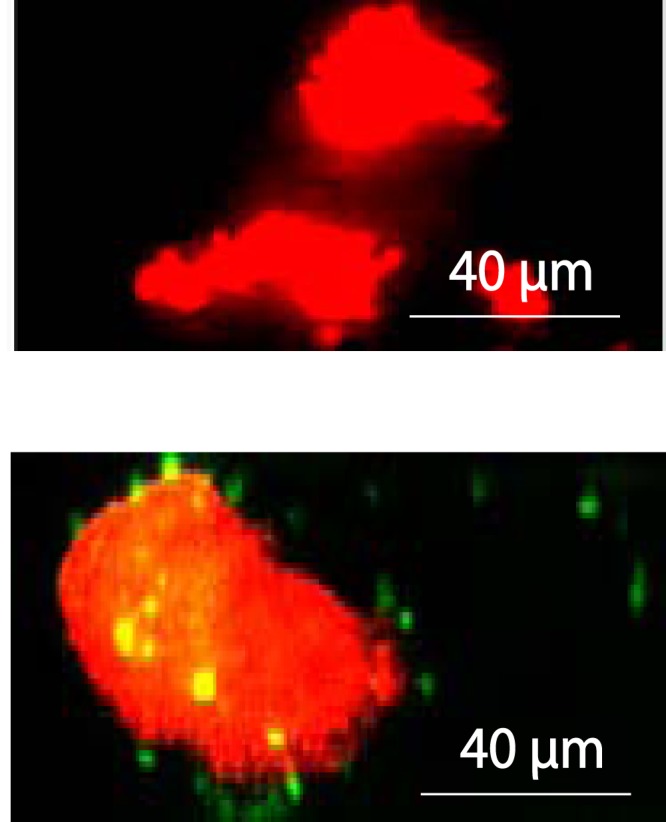
NA stimulates the release of neutrophil extracellular traps (NETs). Microfluidic bioreactors were infused with FITC-labeled primary antibodies specific to human citrullinated histone H3 and 1 µg/ml PI 20 h after coculture of B. thailandensis cells with human neutrophils. Shown are overlay fluorescence microscope images of a B. thailandensis-neutrophil coculture not treated with NA (top) and neutrophils pretreated with 100 µM NA prior to bacterial infection and subsequently incubated in medium containing 25 µM NA (bottom). Cells were imaged at excitation and emission wavelengths of 488 and 540 nm, respectively, for green staining and excitation and emission wavelengths of 520 and 610 nm, respectively, for red staining. Histone H3 is seen as green or yellow overlay staining.

We also examined whether the neutrophil-mediated bactericidal effect was in part due to the NA-stimulated release of neutrophil extracellular traps (NETs). NET formations consist of chromatin enmeshed with granular and nuclear proteins and are known to capture and kill microbial cells (21). We infused the microfluidic devices with antibodies specific to human citrullinated histone H3, a major protein component of the NETs (22), and detected antibody-labeled puncta (green and yellow overlay stain) against the NET-like nucleic acid released formations (red stain) in samples supplemented with NA but not in bacterium-neutrophil cocultures not treated with NA (Fig. 4).

### Immunoprophylaxis with NA exacerbated the outcome of infection in BALB/c mice exposed to B. pseudomallei K96243.

**FIG 5:**
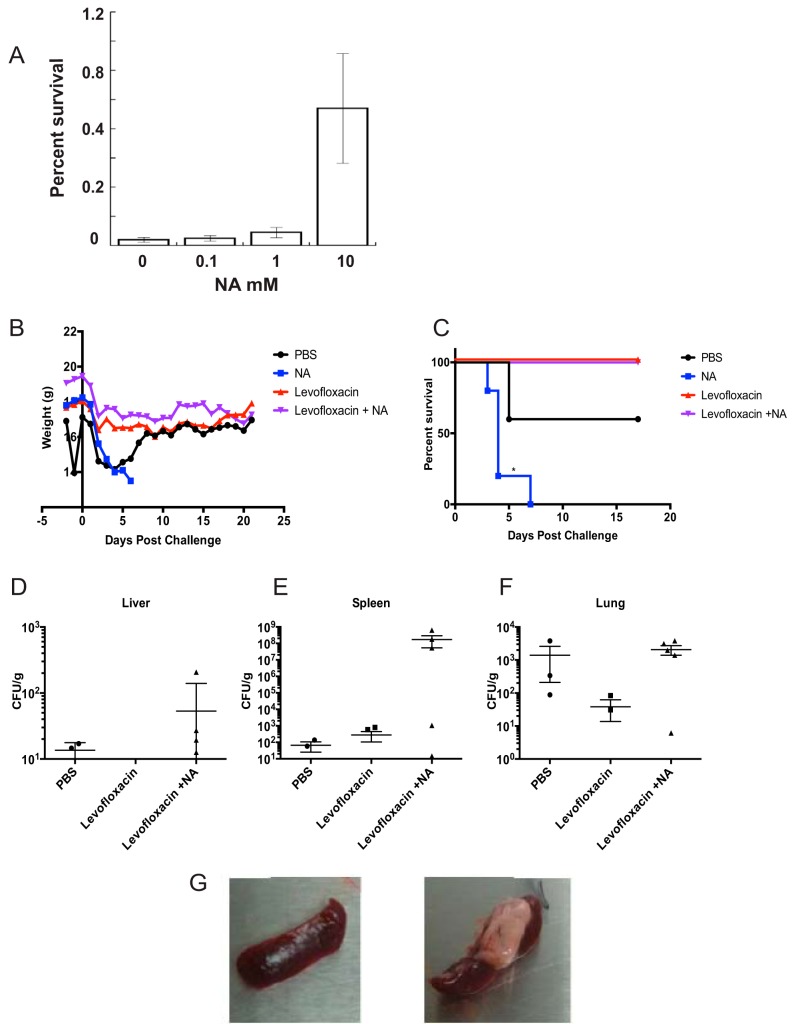
Immunoprophylaxis with a high dose of NA resulted in increased host mortality and enhanced persistence of B. pseudomallei in target organs. (A) A high dose of NA increased the survival rate of B. pseudomallei K96243 in response to levofloxacin. Stationary-phase bacterial cultures of B. pseudomallei K96243 were diluted 2-fold with fresh LB containing 100× MIC levofloxacin or a combination of antibiotic and NA at the indicated concentrations. Bacterial cultures were incubated for 24 h at 37°C with no aeration. Percent survival was determined as the number of CFU per milliliter of antibiotic-treated cells versus the bacterial counts prior to antibiotic treatment. The statistics were obtained from 6 experimental replicates. Mice (*n* = 5) were pretreated with NA or PBS at 2 days prior to infection with 2 LD_50_ of B. pseudomallei K96243. After infection the mice received either PBS, continued NA treatment, levofloxacin, or levofloxacin in combination with NA starting at 24 h following infection and lasting 5 days. (B, C) Through the course of infection, weight (B) and survival (C) were monitored. (D to F) At 21 days postinfection, the liver (D), spleen (E), and lungs (F) were collected for enumeration of the CFU of residual bacteria. (G) Gross histopathology differences between mice treated with levofloxacin (left) or levofloxacin in combination with NA (right) are displayed. Survival curves were analyzed by the log-rank test, and statistically significant difference between treatments are indicated (*, *P* < 0.05).

We performed *in vitro* studies with Burkholderia pseudomallei K96243 prior to testing the effect of NA as a therapeutic supplement *in vivo*. NA was added to stationary-phase cultures in combination with 100× MIC levofloxacin treatment. The antibiotic potency against persisters was examined at 24 h posttreatment, when the bactericidal effect reached a plateau (16). We observed no significant effect on the potency of levofloxacin against persisters at the lower concentrations of 0.1 and 1 mM NA. However, a high dose of 10 mM NA markedly increased the survival rate of B. pseudomallei when it was combined with levofloxacin (Fig. 5A).

To determine whether NA could function as a therapeutic agent to augment antibiotic killing of persisters and promote more effective immune-mediated killing, we applied NA as a prophylactic agent prior to infection with Burkholderia pseudomallei K96243 in a murine model of melioidosis. Both B. thailandensis and B. pseudomallei share significant genetic similarity and resistance to various classes of antibiotics linked to efflux pump and β-lactamase activities (23, 24). We based our treatment regimen on the previous successful use of NA against bacterial infections in mice (13, 14) and on our *in vitro* data showing a stronger effect on persister eradication when NA was introduced prior to antibiotic treatment. We administered NA at 250 mg/kg of body weight or phosphate-buffered saline (PBS) to BALB/c mice daily for 2 days and then exposed them to 2 50% lethal doses (LD_50_) B. pseudomallei via the intranasal route. Antibiotic treatment using levofloxacin alone or in combination with NA was initiated at 24 h after bacterial infection and lasted for 5 days. Two control groups, PBS-treated mice and NA-treated mice, did not receive antibiotic.

Strikingly, the control group treated only with NA exhibited the most adverse response to B. pseudomallei infection, showing irreversible weight loss and ruffled fur (severe clinical signs), and 100% of the animals succumbed to infection within 7 days (Fig. 5B and C). Severe clinical signs were also observed in half of the control animals receiving PBS. Greater than 50% of the PBS-treated animals showed recovery past the 5th day of infection and survived up to 21 days postinfection, when the experiment was terminated. Consistent with the adverse effects of the NA treatment, we recovered 10-fold and 10^6^-fold higher numbers of CFU of B. pseudomallei in the liver and the spleen, respectively, for animals that received the NA-levofloxacin combination than for animals that received only PBS (Fig. 5D and E). The bacterial counts in the lungs of animals treated with NA-levofloxacin were similar to those in the lungs of the PBS-treated control group, and the bacterial counts in the lungs of animals in both of these groups were 10-fold higher than the counts of live bacteria in the lungs of animals treated with just levofloxacin (Fig. 5F). Furthermore, at 21 days postinfection and prior to termination of the experiment, we observed that the group treated with NA-levofloxacin started to show symptoms of infection resurgence, which could have been due to the very high bacterial counts in the spleen. Lobulated yellowish lesions were observed only in the spleen of the animals treated with the combination of NA-levofloxacin that survived until the end of the experiment (Fig. 5G). These formations are indicative of the granulomatous inflammation caused by activated epithelioid macrophages (25). Altogether, our *in vivo* results show that a high dose of NA led to an increase in B. pseudomallei spread and colonization of target organs in a murine infection model.

## DISCUSSION

In addition to genetic inheritance of antibiotic resistance, many microbes also display bacterial persistence or noninherited antimicrobial resistance, in which a small percentage of the cell population (0.01% to 0.1% of E. coli bacteria in log-phase growth) enter a nearly dormant metabolic state and are subsequently refractory to antibiotic killing (6, 15). Metabolite-based strategies to kick-start persisters into a metabolically active state to potentiate antibiotic activity against persisters may significantly improve the outcome of chronic bacterial infections. In this study, we found that persistence occurred at comparatively higher frequencies in B. thailandensis (0.1% to 1% of bacteria in log-phase growth), thus making *Burkholderia* an excellent bacterial species to further study persistence.

We observed that different antibiotics exhibited varied potency in killing persisters. For example, Oflx, which targets DNA gyrase, was more effective at killing persisters than Tmp or Ceftz, which target dihydrofolate reductase and cell wall synthesis, respectively. Although the potency of antibiotics against the persister population was varied, as evidenced by the percentage of cell survival, the phenomenon of persistence was universal since no antibiotic alone was capable of completely eradicating the persister population. The varied survival rates of persisters in response to antibiotics with different mechanisms of action indicate that there are different pathways to establish persistence. Interestingly, exposure to a minimum bactericidal dose of antibiotics led to a boost in ATP accumulation in the first 3 h, suggesting that antibiotics can initially stimulate energy generation. Antibiotic treatment has been reported to stimulate energy-consuming secondary metabolic pathways and activation of efflux pumps, establishing a link between bactericidal lethality and antibiotic-induced cellular respiration (26, 27). Bacteriostatic antibiotic treatment of E. coli and S. aureus has been shown to lead to accumulation of energy metabolites that feed the electron transport chain (26). Thus, we speculate that the tipping point for whether a bacterial cell dies or becomes persistent in response to antibiotics is dependent on the stimulated level of energy generation in each individual cell.

We also demonstrate that the metabolite NA could potentiate the bactericidal effect of different antibiotics against B. thailandensis *in vitro*. NA is a precursor of NAD, a coenzyme in the glycolysis and the Krebs cycle metabolic pathways. NAD is transformed into NADH by accepting electrons in several reactions involved in energy metabolism. For each pair of electrons passed along the electron transport chain, one NADH molecule is used and three ATP molecules are generated. NA was also shown to inhibit poly-ADP-ribose polymerase, which is responsible for NAD depletion by transferring poly-ADP-ribose subunits from NAD to various DNA repair enzymes (28). Our finding that NA also inhibited the transcription of *relA* in B. thailandensis during stationary phase is consistent with NA acting to stimulate persister growth. RelA is a key regulator of the stringent response during times of stressful environmental conditions, allowing bacteria to rapidly divert energy and resources toward processes that ensure cell survival (29–31).

In previous studies, NA was shown to significantly relieve disease symptoms in mouse models of Parkinson’s and Huntington’s diseases (32, 33). NA has been used as an immunomodulation agent to suppress secretion of proinflammatory cytokines and chemokines (34–36) and to improve host survival during murine models of sepsis (35, 37). Furthermore, NA has been tested as an antimicrobial therapeutic agent in murine models of infection caused by opportunistic pathogens, including Staphylococcus aureus and Citrobacter rodentium, and was found to stimulate host neutrophil cells to release microbicidal peptides by direct activation of the myeloid-specific transcription factor CCAAT/enhancer-binding protein ε (C/EBPε) (13, 14). Because NA was found to have a therapeutic effect in previous infection models, we utilized two systems to assess NA-assisted antibiotic killing of *Burkholderia*: (i) microfluidic devices with a continuous flow of nutrient medium to simulate the host circulatory system and (ii) an *in vivo* murine model of B. pseudomallei infection. The microfluidic devices allowed us to generate concentration gradients of NA to assess the B. thailandensis response to antibiotic treatment in a single device. We observed that while high doses of NA (∼100 μM) had a bacteriostatic effect and may have contributed to the increase in the percentage of persister cells, lower NA levels (<25 μM) combined with human neutrophils was the most efficacious regimen tested for the elimination of B. thailandensis persisters. We also observed that human neutrophils pretreated with NA efficiently released a meshwork of chromatin fibers, structures known as neutrophil extracellular traps (NETs), when in coculture with B. thailandensis. In addition to the chromatin fibers, NETs consist of antimicrobial peptides and enzymes and are considered an important strategy to trap and kill microorganisms (38–40).

Our initial success using NA to eliminate persisters in the microfluidic devices led us to test NA as a prophylactic treatment using an *in vivo* murine model of B. pseudomallei infection. Although NA at low concentrations (0.1 and 1 mM) did not induce a significant change in B. pseudomallei persister survival in response to levofloxacin when added to stationary-phase cultures *in vitro*, a high, 10 mM NA concentration led to increased B. pseudomallei persister survival. To test these results in *in vivo* studies, we applied the therapeutic concentration of NA, 250 mg/kg, previously used for *in vivo* animal studies of bacterial infection. For comparison, the maximum daily tolerated dose of NA in humans is 6 g, with the average maximum recorded plasma level (mean ± 1 standard error of the mean) being 156.4 ± 33.6 µg/ml (41), which is about half the dose in the murine studies. While NA had stimulated immune-mediated killing of S. aureus and C. rodentium in murine models (13, 14), we observed an extremely adverse effect in response to infection of BALB/c mice with 2 LD_50_ of B. pseudomallei K96243 receiving 250 mg/kg/day NA administered alone. There was 100% mortality in the animal group that received NA as a prophylactic treatment with no antibiotic supplementation, whereas the rate of mortality in the group treated solely with saline solution was 50%. Given that NA has been reported to enhance neutrophil killing of bacteria in mice (13) and increase the number of neutrophils in the peripheral blood of humans (42), we hypothesize that NA may hyperstimulate the host immune response, specifically, the neutrophil function, leading to rapid animal death upon B. pseudomallei infection. Interestingly, adverse immune responses were not reported when 250 mg/kg/day NA was used in the S. aureus and C. rodentium mouse infection studies.

It is likely that the NA concentration is a key determinant whose potentiation effect varies depending on the metabolism of the specific bacterial species. Our studies suggest that the effect of NA as a metabolite whose activity is auxiliary to the antibiotic activity against persister populations may be a general phenomenon in some species. We have also observed that NA is capable of potentiating antibiotic activity against Pseudomonas aeruginosa under experimental conditions similar to those of our B. thailandensis studies (data not shown). Although we found that a high dose of NA was detrimental for animals during infection with B. pseudomallei, it may be the case that lower doses of NA (e.g., 5 to 10 mg/kg/day) continuously administered using an optimized route following infection could improve antibiotic efficiency against persister cells. It was previously shown that 40 µM NA could inhibit interleukin-1β (IL-1β), IL-6, and tumor necrosis factor alpha cytokine responses by >95% in human whole-blood cells stimulated *in vitro* by endotoxin (34). Inhibition of systemic inflammatory responses has the potential to increase the survival outcome from lethal B. pseudomallei infections. For example, immunomodulation via the COX-2 inhibitor significantly improved antimicrobial treatment efficacy in BALB/c mice after lethal pulmonary infection with B. pseudomallei, resulting in a lower bacterial burden in the spleen (43). The testing of lower doses of NA or alternative routes of administration as a prophylactic treatment of *Burkholderia* infection could be the focus of future studies.

Due to the rise of antibiotic resistance among pathogenic bacteria, there is growing interest toward developing therapeutic approaches based on the modulation of the host immune response. The results from our study caution us to carefully evaluate each therapeutic approach involving immunomodulation and to tailor it to the specificities of the immune response to each pathogen, since there is great variance in the outcome of host-pathogen interactions.

## MATERIALS AND METHODS

### Bacterial strains.

B. thailandensis CDC2721121 cultures were grown in LB medium overnight at 37°C. Antibiotic sensitivity tests were performed in LB medium at 37°C with aeration or in microfluidic bioreactors with a continuous flow of LB medium. B. pseudomallei K96243 (BEI Resources, Manassas, VA, USA) was used in the murine infection experiment. B. pseudomallei was propagated on LB agar and then LB medium at 37°C. All manipulations of B. pseudomallei were conducted in compliance with the select agent regulations and in CDC/USDA-approved and registered biosafety level 3 (BSL3) facilities at the University of Texas Medical Branch (UTMB).

### Human tissue culture.

Normal human neutrophils (NHN) were obtained from Astarte Biologics (catalog number 1025; Bothell, WA) and cultured in RPMI medium (Invitrogen, Carlsbad, CA) supplemented with 5% fetal bovine serum and 2 mM l-glutamine in a 5% CO_2_ humidified incubator at 37°C for 24 h prior to experimental manipulations. For the 24-h pretreatment of human neutrophils, NA was added immediately after thawing of the cells, since we observed clumping of NHN cells and decreased viability after 40 h of incubation, as measured by trypan blue staining.

### Determination of MIC and bacterial persistence fraction.

MICs for antibiotics were determined in LB medium by microdilution following standard CLSI (formerly NCCLS) protocols. The MICs were determined as the antibiotic concentration at which the optical density at 600 nm (OD_600_) remained unchanged within 24 h of bacterial culture compared to the seeding density. Ceftz (catalog number C0690500), Mpm (catalog number 392454), Tpm (catalog number T7883), and Oflx (catalog number 1478108) were obtained from Sigma-Aldrich (St. Louis, MO). To determine the population of persister cells, we inoculated 2 ml Luria broth (Miller, Fisher Scientific) in 5-ml Falcon tubes with one colony of B. thailandensis from an LB agar plate, and the bacteria were incubated at 37°C with low aeration at 120 rpm until the OD_600_ reached 0.2, prior to addition of antibiotics for the samples in exponential growth phase. To test the effect of NA on the antibiotic-mediated bactericidal effect, the metabolite was supplemented in the culture medium prior to addition of antibiotics. Limiting dilutions of bacterial cultures prior to addition of antibiotic were plated on antibiotic-free LB agar plates to determine the bacterial counts at the 0-h time point as the number of CFU per milliliter. Stationary-phase samples originated from overnight bacterial cultures incubated in medium supplemented with NA without aeration, and medium with antibiotic was added at a 1:1 dilution ratio. The percent survival of persisters was determined after 24 h of antibiotic treatment. *In vitro* experiments with B. pseudomallei cultures were performed without NA supplementation prior to antibiotic treatment. Stationary-phase B. pseudomallei bacteria were adjusted to 1 × 10^8^ CFU per well and treated with 100× MIC (400 µl) of levofloxacin or ceftazidime in combination with NA at a final concentration of 0, 0.1, 1, or 10 mM. At 24 h after antibiotic treatment, bacteria were collected, resuspended in fresh medium, and plated for CFU enumeration. The number of surviving bacteria was normalized by the input and displayed as percent survival. The number of CFU per milliliter of culture was determined by plating serial dilutions of bacterial cells on antibiotic-free LB agar plates and counting the colonies after 24 to 48 h of incubation at 37°C. The percent survival of drug-tolerant persister cells was determined by exposing bacterial cultures to antibiotics at 200× MIC for Ceftz and Tpm and 100× MIC for Mpm and Oflx. After antibiotic treatment, bacterial cultures were pelleted and resuspended in 1 ml antibiotic-free LB medium. Percent survival was determined as the viable CFU counts following 10-fold serial dilution in LB medium and plating on LB agar as a fraction of the CFU count of the input culture. All assays were performed in triplicate. Tests for genetically acquired antibiotic resistance were performed by plating bacterial cultures surviving antibiotic treatment on LB agar plates containing antibiotic at 50× MIC.

### ATP pool determination.

We applied a BacTiter-Glo microbial cell viability assay (Promega) to measure the ATP content in bacterial samples immediately after obtaining OD_600_ values at particular time points of bacterial culture growth and/or antibiotic treatment. The assay was performed in a 96-well format, with a 1:1 ratio of BacTiter-Glo reagent being adding to the bacterial samples (100 µl). Triplicate measurements of the number of relative light units (RLU) per sample were performed, and average values were calculated.

### Isolation of bacterial RNA and RT-quantitative PCR.

Total RNA was isolated from bacterial pellets preincubated in 1,000 U lysozyme for 15 min at 37°C and dissolved in the TRIzol reagent (1 ml reagent per 10^8^ bacteria). Chloroform was added to the TRIzol reagent extract (1:4 ratio), and samples were incubated at room temperature for 5 min. The aqueous phase containing RNA was separated from the organic phase by centrifugation for 15 min at 12,000 × *g* (8°C). An equal volume of 100% chilled ethanol was added to the RNA solution and then loaded on an RNA-binding column from an miRNeasy kit (Qiagen). RNA was eluted in water, and residual genomic DNA was removed by treatment with a Turbo DNA-free kit (Thermo Fisher Scientific). The RNA concentration was determined using an ND1000 NanoDrop spectrophotometer (Thermo Scientific), and RNA quality was evaluated on a denaturing 1% agarose gel. A Power SYBR green RNA-to-Ct one-step kit (Thermo Fisher Scientific for Applied Biosystems) was used to perform reverse transcription (RT)-PCR for transcript calculations. For each RT-PCR, the following transcript-specific forward and reverse primers (final concentration, 600 pmol) were used: RelA_fw (5ʹ-AAT TCC TCG AGC ACG TGA AG), RelA rev (5ʹ-GAG ATC GCT GTG GAT CGA ATA C), dnaJ_fw (5ʹ-CAG GGC TTC TTC AGC ATT CA), dnaJ_rev (5ʹ-GTG AAG GAA ACC AAG ACG CT), 16S/rrsD_fw (5ʹ-AGG CCT TCG GGT TGT AAA G), and 16S/rrsD_rev (5ʹ-GTA GTT AGC CGG TGC TTA TTC T). The RT-PCRs were performed on an ABI 7500 Fast system (Applied Biosystems) with cycling conditions according to the manufacturer’s protocol for the Power SYBR green RNA-to-Ct one-step kit. *relA* gene transcript levels were normalized to the 16S rRNA level. *dnaJ* transcripts were used as a second normalization control since their abundance was comparable to the level of *relA* expression and remained unchanged under all test conditions. RNA ratios were calculated relative to the RNA level in the control sample, growing B. thailandensis cultures with no treatment, by applying the ΔΔ*C_T_* threshold cycle (*C_T_*) method. RT-PCR products for each transcript were validated via sequencing.

### RNA-FISH experiments for single-cell analysis.

An RNA hybridization probe set for *relA* mRNA consisting of 40 Quasar 560-labeled antisense oligonucleotides was purchased from Biosearch Technologies utilizing the company’s probe designer tool. The probe set was diluted to 100 μM in nuclease-free water and aliquoted in 5-μl batches for subsequent experiments. Bacterial pellets from exponentially dividing cells (OD_600_, ∼0.4) and stationary-growth-phase cultures (OD_600_, ∼1.2) were collected by centrifugation at 6,000 × *g* for 5 min and fixed in 3.7% formaldehyde for 60 min at room temperature, followed by overnight permeabilization in 70% ethanol at 4°C. Fixed and permeabilized bacterial samples were washed once with 2× saline sodium citrate (SSC; 1× SSC is 0.15 M NaCl plus 0.015 M sodium citrate) buffer containing 2% deionized formamide and resuspended in 100 μl of hybridization buffer (1 g dextran sulfate, 10 mg E. coli tRNA, 100 μl of 200 mM vanadyl ribonucleoside complex, 40 μl of 5 mg/ml bovine serum albumin, 1 ml 20× SSC, 2 ml deionized formamide) with a final volume of 10 ml. The probe set was diluted in hybridization buffer at a 1:100 ratio to a final concentration of 1 μM. Samples were incubated overnight at 30°C in the dark. The bacteria were then pelleted by centrifugation and washed twice with 500 μl of 2× SSC buffer containing 2% formamide and 2 ng/ml DAPI (4′,6-diamidino-2-phenylindole) using 30-min incubations at 30°C. Cell samples were finally resuspended in 100 μl of 2× SSC buffer. Imaging was performed on an Olympus IX71 inverted microscope, with excitation provided by a filtered mercury arc lamp. Data processing was performed with custom Matlab code as previously described (44), with slight modifications for bacterial cell identification using images collected with the DAPI filter.

### Microfluidic chip fabrication and continuous cell culture.

The microfluidic device consisted of 33 mixing channels ascending from 3 to 8 per row and merging into a single-cell growth chamber (3.4 by 1 mm in length and width) (Fig. 2A). Each cell growth chamber contained an array of 18 (6 by 3) cell trapping chambers (diameter, 40 μm). The lithography photomask with the desired chip layout was produced in-house by a 2-μm process control marker (PCM) with a tolerance of ±0.25 μm following previously established protocols (18). The polydimethylsiloxane (PDMS) chip fabrication base and curing reagent were mixed at a 10:1 ratio to a homogeneous solution and degassed in a vacuum chamber for 15 min or until all bubbles disappeared. The degassed PDMS mixture was poured over an SU-8 wafer with the designed microfluidic pattern and cured for 3 h on an 80°C hot plate or overnight at room temperature. Before mounting the PDMS chip on a glass slide, the inlet and outlet holes were punched out with a 40-µm needle, cleaned with isopropanol, and plasma treated at 50 W for 300 s. The PDMS and glass chip were aligned and bonded for 30 min at 60°C. Prior to inoculation of the microfluidic chip with bacterial or human cells, a 1-ml syringe was attached to tubing and 70% ethanol was run through the microfluidic chip for 2 h at a 50-µl/h speed using a syringe pump, followed by a sterile water wash for 1 h and infusion with growth medium for 2 h at 50 µl/h. Bacterial cells were delivered into the growth chamber through a seeding inlet with a 1-ml syringe using a syringe pump, and the fluid was run through the chamber for 5 min. When the desired cell density was achieved, the cell seeding inlet was clamped and the microfluidic chip was mounted in a microscope incubator preheated to 37°C. The microscope chamber was connected to 5% CO_2_ through a gas flow regulation system when human neutrophils were inoculated in the microfluidic device.

### Fluorescence analysis.

Bacterial and human neutrophil cell death was observed with propidium iodide (PI), added to the cell culture medium at 1 µg/ml and infused into the microfluidic growth chamber after termination of antibiotic incubation. For imaging of neutrophil extracellular traps (NETs), antibody against human citrullinated histone H3, H3CitArg2/8/17 (catalog number LS-C144555; LifeSpan Biosciences, Seattle, WA), was linked to fluorescein isothiocyanate (FITC) via a Lightning-Link fluorescent antibody labeling kit (catalog number 707-0010; Novus Biologicals, Littleton, CO). FITC-labeled anti-H3CitArg2/8/17 was added to LB medium at a 1:300 dilution and infused into the microfluidic chip through the medium inlets for 18 h at room temperature and a 2-µl/h flow speed. LB medium alone was supplied for another 18 h to wash the unbound antibody, and florescent images were taken with a Zeiss Axio Observer Z1 microscope at 520- and 610-nm excitation and emission wavelengths, respectively, to capture nucleic acid staining and 488- and 520-nm excitation and emission wavelengths, respectively, to capture citrullinated histone H3 staining.

### Infection of mice and determination of tissue bacterial load.

The animal studies were carried out in strict accordance with the recommendations in the *Guide for the Care and Use of Laboratory Animals* of the National Research Council (45); U.S. Department of Defense instruction 3216.01, dated September 2010; and Army regulation 40-22. The experimental protocol was approved by IACUC (IACUC number 0503014D) at the University of Texas Medical Branch (UTMB) and the Animal Care and Use Review Office (ACURO) at the U.S. Army Medical Research and Materiel Command.

Female BALB/cJ mice (age, 6 weeks), obtained from The Jackson Laboratory, were used. The mice were allowed to acclimate for 5 days prior to infection. Anesthetized BALB/c mice (*n* = 5 per treatment) were inoculated intranasally (i.n.) with 2 LD_50_ B. pseudomallei K96243, diluted in phosphate-buffered saline (PBS) in a total volume of 50 µl (25 µl/nare). Mice received daily intraperitoneal injections of levofloxacin (25 mg/kg/day in PBS) starting at 24 h postinfection and continuing for 5 days. Mice were monitored daily for weight changes and survival over a period of 20 to 21 days. For CFU enumeration, animals were euthanized and their lungs, livers, and spleens were collected for CFU enumeration and homogenized using Covidien Precision tissue grinders (Fisher Scientific). Tissue homogenates were serially diluted in PBS, plated, and incubated for 48 h at 37°C. Colonies were counted and normalized to organ weight (in grams).

### Statistics.

For the *in vitro* data on the antibiotic effect on bacterial survival, we applied a paired Student’s *t* test. Survival curves were analyzed by using the Kaplan-Meier method. A significant difference (*P* ≤ 0.05) in survival curves was ascertained via a log-rank test. CFU enumeration significance was determined using a *t* test with a Mann-Whitney correction. We considered a *P* value below 0.05 to be significant.

## Supplementary Material

Supplemental file 1

Supplemental file 2

Supplemental file 3
