# Supplementary figures and images for: Increased Mortality in Mice following Immunoprophylaxis Therapy with High Dosage of Nicotinamide in Burkholderia Persistent Infections
Source: Infect Immun. 2018 Dec 19;87(1):e00592-18. doi: 10.1128/IAI.00592-18 (PMC6300628; doi:10.1128/IAI.00592-18)

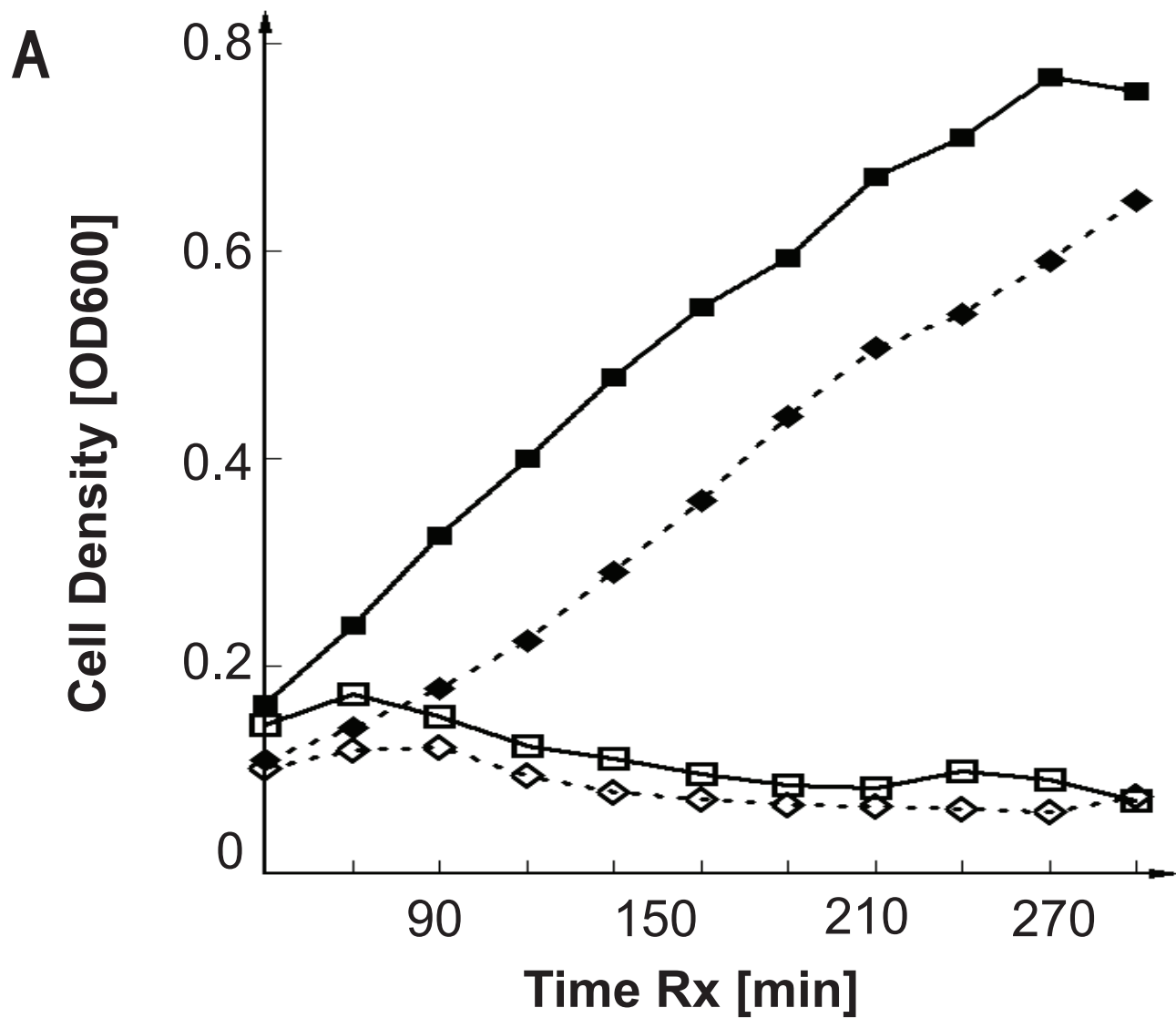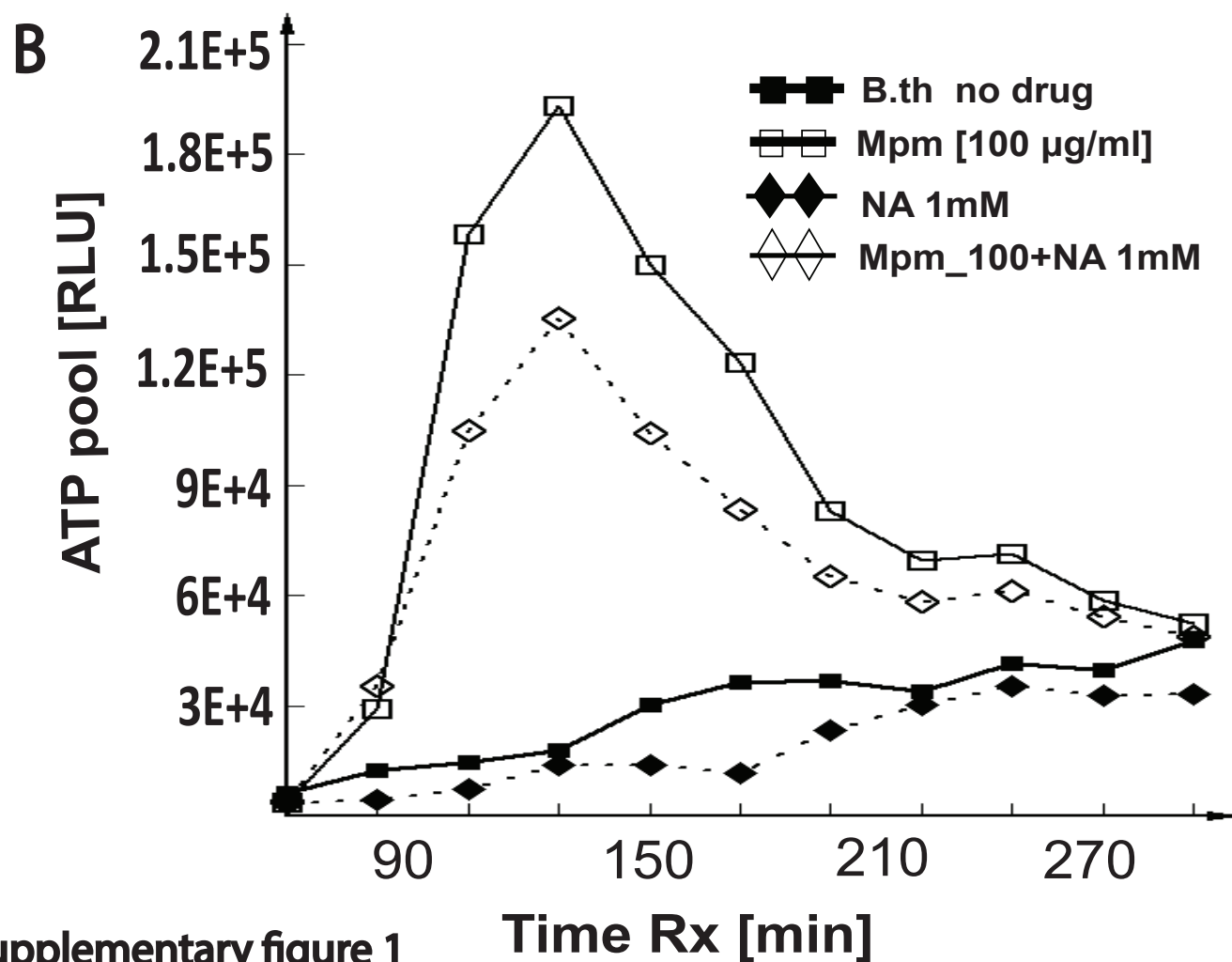

Supplementary figure 1

Supplement: Supplemental file 1 [file d48b3b1a28cf221e908381c4142586f2_IAI.00592-18-s0001.pdf]

A

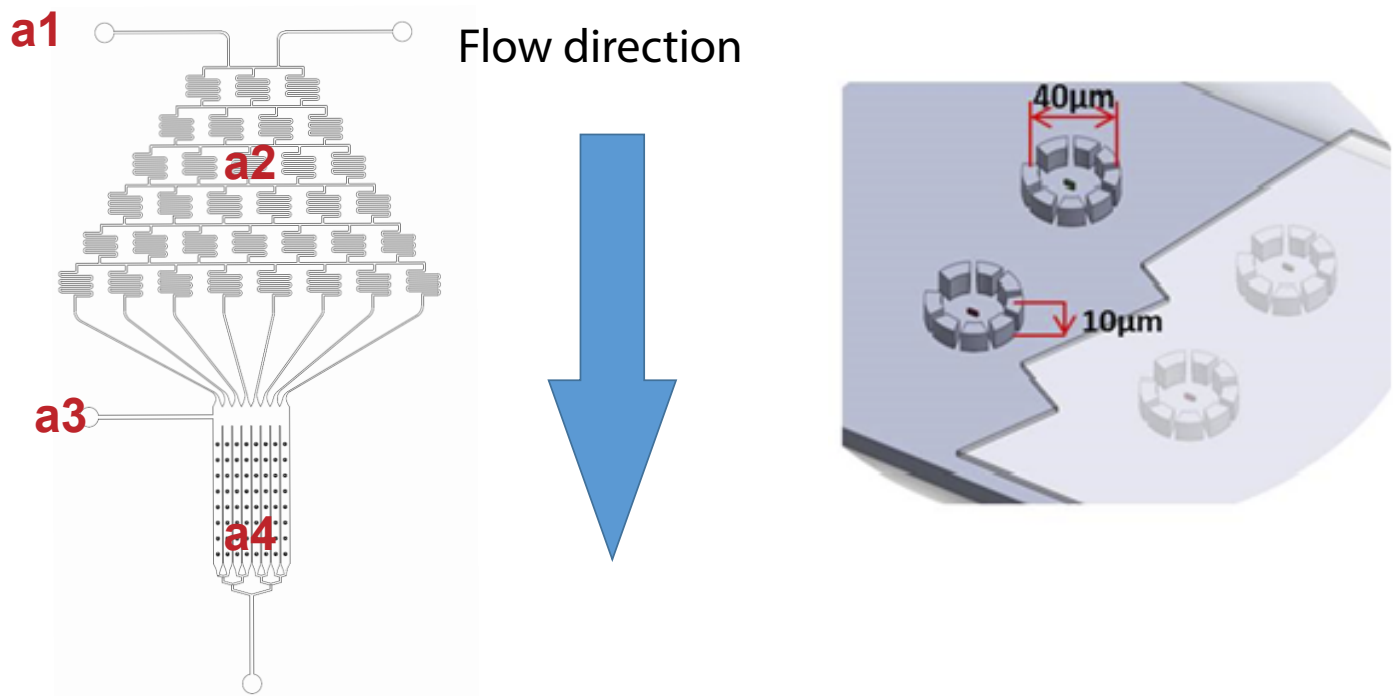

B

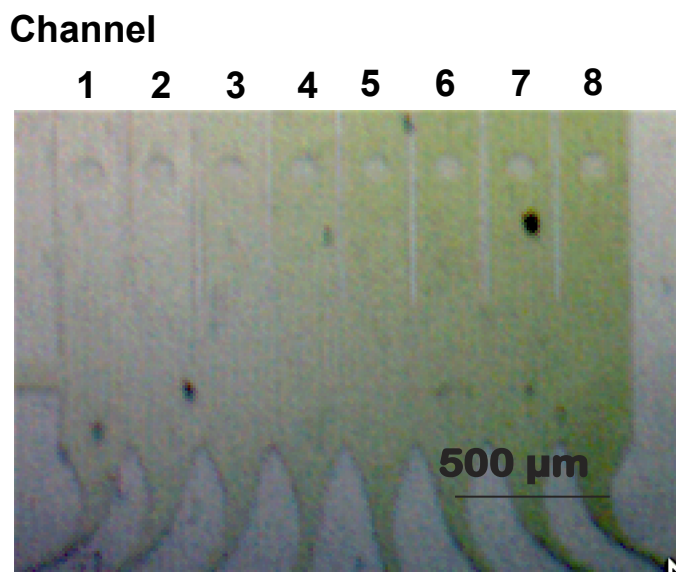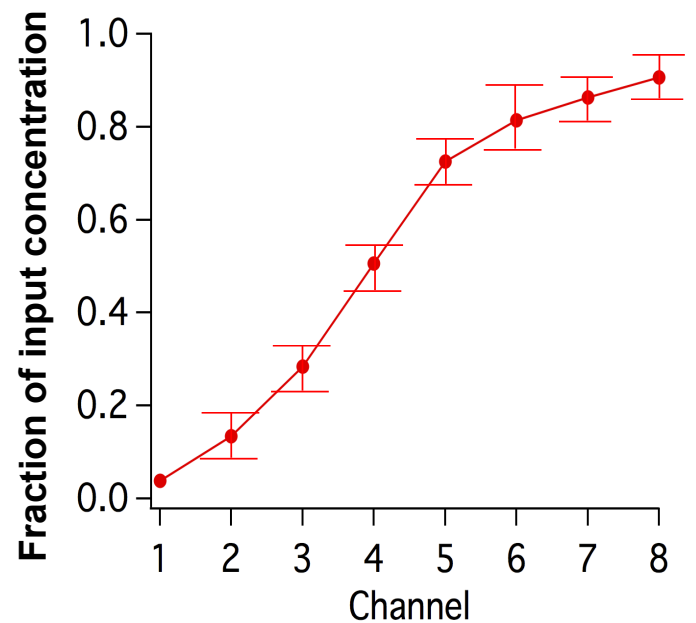

Supplementary Figure 2

Supplement: Supplemental file 2 [file 41c4f54c90bab6f8479d10638613ca75_IAI.00592-18-s0002.pdf]
