# Supplementary material for: Increased Mortality in Mice following Immunoprophylaxis Therapy with High Dosage of Nicotinamide in Burkholderia Persistent Infections
Source: Infect Immun. 2018 Dec 19;87(1):e00592-18. doi: 10.1128/IAI.00592-18 (PMC6300628; doi:10.1128/IAI.00592-18)
Supplement: Supplemental file 3 [file 2fe7cef31920071a9e57c5e1c98940f6_IAI.00592-18-s0003.pdf]

Fig. S1. ATP pool increase in antibiotic-treated *B. thailandensis*. *B. thailandensis* cells were treated at the exponential phase with 100µg/ml Mpm, 1mM NA, or a combination of antibiotic with NA. Samples were analyzed at 30 min intervals for cellular density by measuring OD_{600} (A) and ATP content (B) applying BacTiter Glo reagent (Promega). RLU, relative light units. A representative of three independent experiments is shown.

Fig. S2. Use of microfluidic bioreactors to study the effect of NA on bacterial sensitivity to antibiotic treatment. (A) Design of the microfluidic chip. Left panel shows layout of the whole microfluidic chip with two inlets for media infusion (a1), a gradient generator with mixing chambers (a2), cell infusion inlet (a3) and cell growth chamber containing array of cell trapping chambers (a4). The right panel shows the design of cell trapping chambers with inner diameter of 40µm and 10 µm depth. **(B) Metabolite concentrations in the microfluidic bioreactor was estimated relative to the concentration gradient of a colorimetric dye.** A syringe pump was used to inject two solute streams into the gradient generator. LB and LB media infused with green dye were supplied through the media inlets (a1) feeding into channel 1 and channel 8, respectively, at speed 10µl/hour. Microscopic images were obtained from channel 1 through 8 in the cell growth chamber (a3). Image pixel number corresponding to color intensity in each channel was used to calculate the fraction of dye input concertation via Igor software application. A representative of 3 independent experiments is shown. Linear correlation between the dye concentration and optical density could be achieved three channels removed from the dye input channel 8. For the channels 1-6 the fraction of input concentration was used to estimate the approximate drug or metabolite concentration.
